# Comparative transcriptome analysis reveals the regulatory mechanisms of two tropical water lilies in response to cold stress

**DOI:** 10.1186/s12864-023-09176-w

**Published:** 2023-02-21

**Authors:** Xiangyu Ma, Qijiang Jin, Yanjie Wang, Xiaowen Wang, Xuelian Wang, Meihua Yang, Chunxiu Ye, Zhijuan Yang, Yingchun XU

**Affiliations:** 1 College of Horticulture, Key Laboratory of Landscape Agriculture, Ministry of Agriculture and Rural Affairs, East China Key Laboratory of Flower Biology, Key Laboratory of Flower Biology and Germplasm Creation, Ministry of Agriculture and Rural Affairs, Nanjing Agricultural University, State Forestry and Grassland Administration, 210095 Nanjing, China; 2 College of Agriculture, Shihezi University, Shihezi, 832000 China; 3 College of Forestry and Horticulture, Xinjiang Agricultural University, Urumqi, 830052 China; 4 Hainan University Sanya Nanfan Research Institute, Sanya, 572000 China

**Keywords:** Cold stress, Water Lily, Phenotypic and physiological changes, RNA-Seq, *NlZAT12*

## Abstract

**Background:**

Tropical water lily is an aquatic plant with high ornamental value, but it cannot overwinter naturally at high latitudes. The temperature drop has become a key factor restricting the development and promotion of the industry.

**Results:**

The responses of *Nymphaea lotus* and *Nymphaea rubra* to cold stress were analyzed from the perspective of physiology and transcriptomics. Under the cold stress, *Nymphaea rubra* had obvious leaf edge curling and chlorosis. The degree of peroxidation of its membrane was higher than that of *Nymphaea lotus*, and the content of photosynthetic pigments also decreased more than that of *Nymphaea lotus*. The soluble sugar content, SOD enzyme activity and CAT enzyme activity of *Nymphaea lotus* were higher than those of *Nymphaea rubra*. This indicated that there were significant differences in the cold sensitivity of the two varieties. GO enrichment and KEGG pathway analysis showed that many stress response genes and pathways were affected and enriched to varying degrees under the cold stress, especially plant hormone signal transduction, metabolic pathways and some transcription factor genes were from ZAT gene family or WKRY gene family. The key transcription factor ZAT12 protein in the cold stress response process has a C_2_H_2_ conserved domain, and the protein is localized in the nucleus. Under the cold stress, overexpression of the *NlZAT12* gene in *Arabidopsis thaliana* increased the expression of some cold-responsive protein genes. The content of reactive oxygen species and MDA in transgenic *Arabidopsis thaliana* was lower, and the content of soluble sugar was higher, indicating that overexpression of *NlZAT12* can improve the cold tolerance of *Arabidopsis thaliana*.

**Conclusion:**

We demonstrate that ethylene signalling and reactive oxygen species signalling play critical roles in the response of the two cultivars to cold stress. The key gene *NlZAT12* for improving cold tolerance was identified. Our study provides a theoretical basis for revealing the molecular mechanism of tropical water lily in response to cold stress.

**Supplementary Information:**

The online version contains supplementary material available at 10.1186/s12864-023-09176-w

## Introduction

Water lily belongs to *Nymphaeaceae* is a world-famous important aquatic plant with high ornamental value, a long flowering period, a wide variety of flowers, and strong adaptability [1]. According to the plant taxonomic system, it is divided into five subgenera, including *Nymphaea*, *Brachyceras*, *Lotos*, *Hydrocallis*, and *Anecphya* [2]. The latter four subgenres are collectively referred to as tropical water lilies. Tropical water lilies are higher than cold-resistant water lilies in terms of ornamental value and economic value. As an indispensable element in garden waterscapes, water lily not only have extremely high ornamental value but can also play a role in purifying water and absorbing heavy metals [3]. The flowers of tropical water lilies are fragrant and are often used to make water lily tea [4]. In some tropical countries, the pedicels and petioles of tropical water lilies are often used as food. The flowers of tropical water lily are rich in polysaccharides, flavonoids and other antioxidant substances, which are often processed into cosmetics, essential oils and medicines [5, 6].

As a major environmental factor, cold stress not only directly affects the growth and development of plants but also indirectly restricts their geographic distribution [7]. When plants are subjected to cold stress, they will experience some changes, such as surface damage, water loss/dryness, tissue rupture, accelerated senescence/ethylene production, FASTer decay and death [8]. Plants generally respond to cold stress by changing their own phenotypes and physiological characteristics, such as activating signal pathways related to cold stress [9] and inducing antioxidant enzymes and membrane systems [10]. The adaptation and potential regulatory system of plants to cold stress includes a comprehensive response involving the regulation of a variety of systems at the biological level, such as genetic regulation, posttranscriptional regulation, posttranslational modification and metabolic feedback [11]. Many low-temperature response-related genes have been identified from different species, such as *Arabidopsis*, rice, and maize [12–14].

Tropical water lilies cannot survive the winter in the northern subtropics (24.3°N north latitude) or northern regions, which has a great impact on the development of the tropical water lily industry and the promotion of varieties [15]. When winter comes, the growth of tropical water lilies introduced to high latitudes will be severely restricted, and they will easily die in cold environments. Many practitioners in the water lily industry will move tropical water lilies into greenhouses or dig deep pits for planting in winter to ensure that the ambient temperature will not affect the survival of tropical water lilies. However, these methods are expensive in terms of labour cost and are not conducive to the growth of tropical water lilies. Therefore, cultivating cold-resistant germplasm of tropical water lilies so that they can naturally overwinter in cold regions is a research hotspot in the field of water lilies, and molecular breeding can solve the pain points of the industry more efficiently than traditional breeding. Exploring the molecular mechanism of the cold stress response and screening key genes related to cold tolerance regulation can provide a theoretical reference for the cultivation of cold-tolerant tropical water lily germplasm. Transcriptome sequencing based on high-throughput sequencing platforms can reveal the global transcriptional activity of any species at the single-nucleotide level [16]. In recent years, transcriptomics has facilitated the study of plant responses to cold stress, and many such studies have been reported [17–19].

According to previous studies, cold-tolerant water lily *Nymphaea lotus* (Nl) and cold-sensitive water lily *Nymphaea rubra* (Nr) were selected as test material, and the morphological changes and some physiological indicators of the two varieties under the cold stress were studied [20]. Then, by comparing the transcriptomes, the differentially expressed genes and pathways of the two varieties in response to cold stress were identified. This study provides a theoretical basis for exploring the molecular mechanism of tropical water lilies in response to cold stress and screening key genes involved in the regulation of cold stress.

## Result

### Physiological and biochemical responses to cold stress

**Fig. 1:**
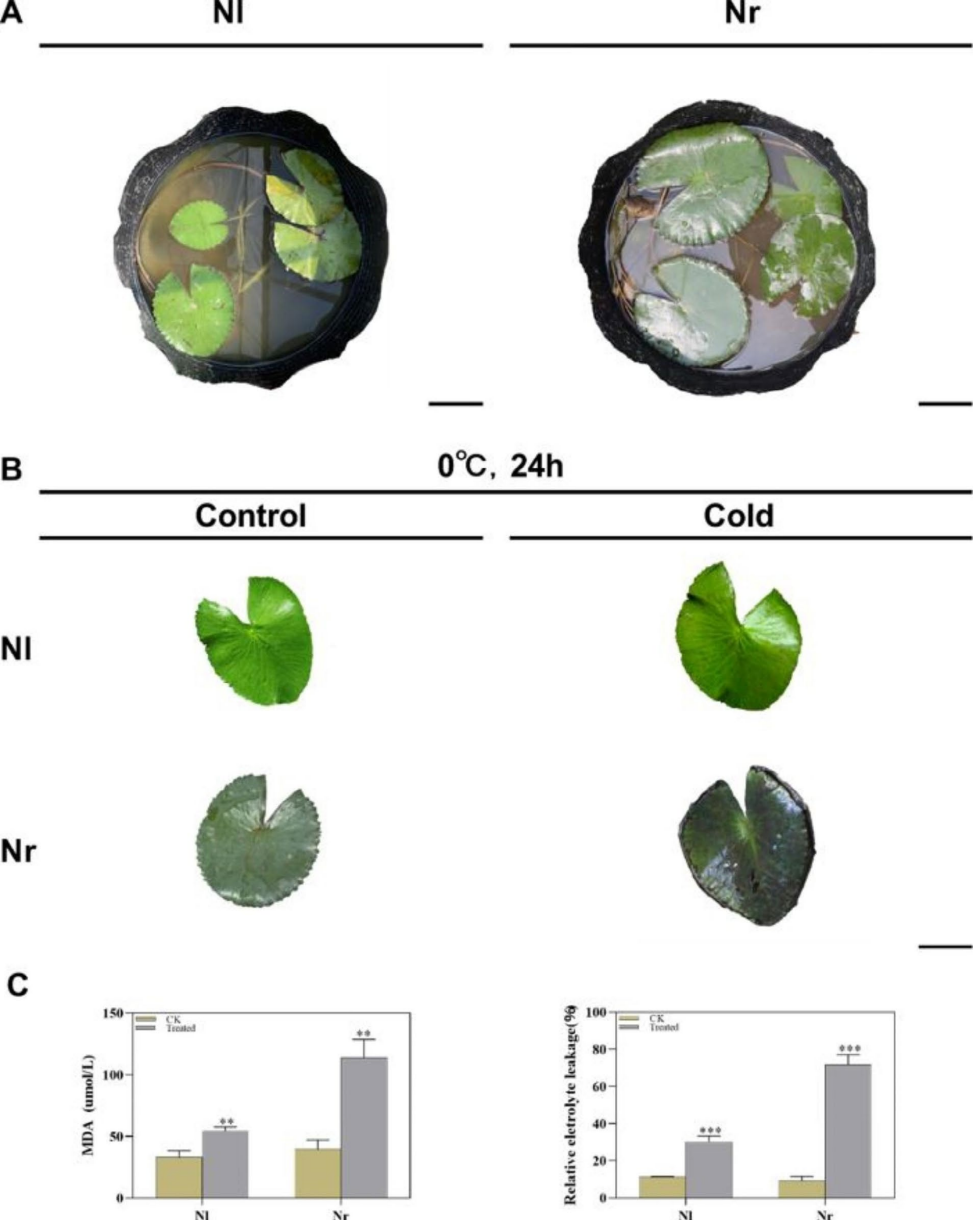
Comparison of the low-temperature sensitivity of Nl and Nr responses. **A**. Growth forms of Nl and Nr in climate chambers (bar = 5 cm). **B**. Phenotypic changes in Nl and Nr after cold stress (bar = 5 cm). **C.** Changes in the relative conductivity and MDA content of Nl and Nr under the cold stress. All data are presented as the mean ± SE from three independent experimental replicates. Asterisks indicate significance determined by t test: ** for p < 0.01 and *** for p < 0.001. Data are shown as the mean of three independent experiments

The plants Nl and Nr in the normal growth state were treated with cold treatment (0 °C) for 24 h, the leaf morphology of Nl did not change significantly, but the leaf morphology of Nr softened and the leaf edge was curled (Fig. 1A and B). Relative electrolyte conductivity (REC) and malondialdehyde (MDA) are commonly used to measure membrane damage and cell stability. Under the cold stress, the membrane system and the balance of ROS metabolism in plant cells are destroyed. After cold treatment, the MDA content of Nl increased by 65% compared with the control group, and the REC increased from 11 to 30%; the MDA content of Nr increased by 187% compared with the control group, and the REC increased from 9 to 72%. Although MDA and REC all increased significantly, but the increase of Nr was more obvious, which indicated that cold stress caused a higher degree of cell damage in Nr (Fig. 1C).

**Fig. 2:**
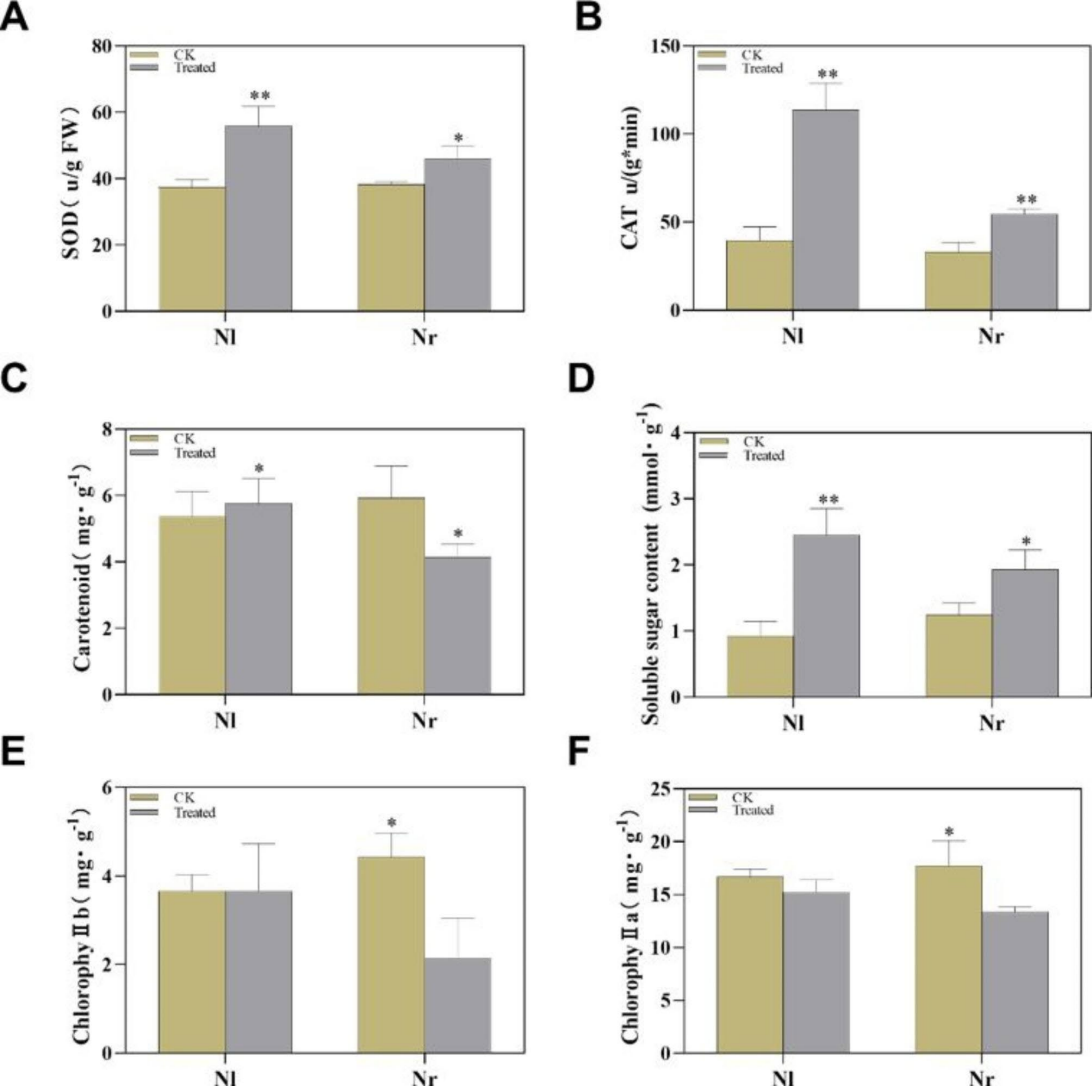
Physiological responses of Nl and Nr under control and cold stress conditions. SOD (**A**), carotene (**B**), CAT (**C**), SS content (**D**), chlorophyll b (**E**) and chlorophyll a (**F**) were measured. All data are presented as the mean ± SE from three independent experimental replicates. Asterisks indicate significance determined by t test: * for p < 0.05 and ** for p < 0.01. Data are shown as the mean of three independent experiments

Previous studies have shown that the tolerance of plants to cold and other adversities is closely related to the concentration of soluble sugar [21]. Under the cold stress, the soluble sugar content increased in the two varieties, but the soluble sugar content in Nl was higher than that in Nr (Fig. 2D). We also determined the chlorophyll content, and the results showed that under the cold stress, the chlorophyll a, b and carotenoid contents of Nr decreased significantly, while Nl did not change significantly (Fig. 2C, E and F). Moreover, we also determined the antioxidant enzyme activities in the leaves of the two genotypes. The results showed that the superoxide dismutase (SOD) and hydroperoxidase (CAT) activities of Nl were significantly improved compared with those of the control conditions. However, in Nr, the activities of SOD and CAT were significantly lower than those in the control conditions (Fig. 2A and B).

### Transcriptomic analyses of Nl and Nr after cold stress

To explore the response to cold stress of two water lilies species, the transcriptome analysis of Nl and Nr after low-temperature treatment were performed. A total of twelve RNA libraries were constructed in this experiment, with three biological replicates for each treatment of both Nl and Nr.

A total of 700 million clean reads were obtained from these libraries after removal of adaptor sequences, low-quality reads (Q-value < 20) and reads with greater than 10% ambiguous N bases. Each library generated approximately 39.8–84.5 million clean reads. Among them, the Q20 of clean reads is 97.86–98.78%, and the Q30 of clean reads is 93.88–95.93% (Additional file 1).

In Nl, using the Trinity program to assemble the clean reads, a total of 118,305 transcripts were generated after clustering. The N50 was 1404 bp, and the GC content was 45.38%. The average length of the transcripts was 888 bp, and their length ranged from 201 to 16,227 bp. In Nr, the Q20% was over 98.02%, and the Q30% was over 94.25%. A total of 114,397 transcripts were generated after clustering. The N50 was 1391 bp, and the GC content was 44.46%. The average length of the transcripts was 902 bp, and their length ranged from 201 to 16,272 bp (Additional file 2).

Quality assessment of assembled transcripts using BUSCO software showed high quality and accuracy of transcriptome assembly (Additional file 3). These data indicated that the assembly was considered high quality and could be used in further analyses.

### Gene annotation and functional classification

For identification of the putative function of these acquired unigenes, the unigene sequence was compared to the protein databases by BLASTX, and the protein with the highest sequence similarity to the given unigene was obtained; thus, the protein function annotation information of the unigene was obtained (Additional file 4).

To further predict unigene function, we searched for all unigenes in the COG database. Based on the COG database, 73,521 unigenes were classified into 25 functional categories. The ‘General function prediction only’ (16,997) cluster represented the largest group, followed by ‘Translation, ribosomal structure and biogenesis’ (10,832), ‘posttranslational modification, protein turnover, chaperones’ (9325), ‘signal transduction mechanisms’ (7678), ‘Energy production and conversion’ (4722), ‘Carbohydrate transport and metabolism’ (3714), ‘RNA processing and modification’ (3430) and ‘Cytoskeleton’ (3392) (Additional file 5 S5 and S8).

GO analyses were used to classify the functions of predicted water lily unigenes. A total of 34,729 unigenes were successfully annotated, Nl had 20,624 unigenes and Nr had 14,105 unigenes (Additional file 5 S6 and S9). All unigenes were classified into three main categories, including biological processes, cellular components, and molecular functions. Metabolic process (20,828), cellular process (16,796), single-organism process (12,815), cell (15,962), cell part (15,951), catalytic activity (17,090), binding (13,286), transporter activity (1714) and structural molecule activity (1596) were the most dominant terms in the three categories.

To identify the active biological pathways in water lily, pathway annotations of the unigenes were performed using the KEGG pathway tool. The KEGG annotated unigenes (95,401) were distributed to 268 KEGG pathways; among these pathways, the ‘metabolic pathways’, ‘ribosome’, ‘biosynthesis of secondary metabolites’, ‘carbon metabolism’ and ‘protein processing in endoplasmic reticulum’ pathways were the most abundant (Additional file 5 S7 and S10).

### Identification of homologous gene families and differentially expressed genes (DEGs)

**Fig. 3:**
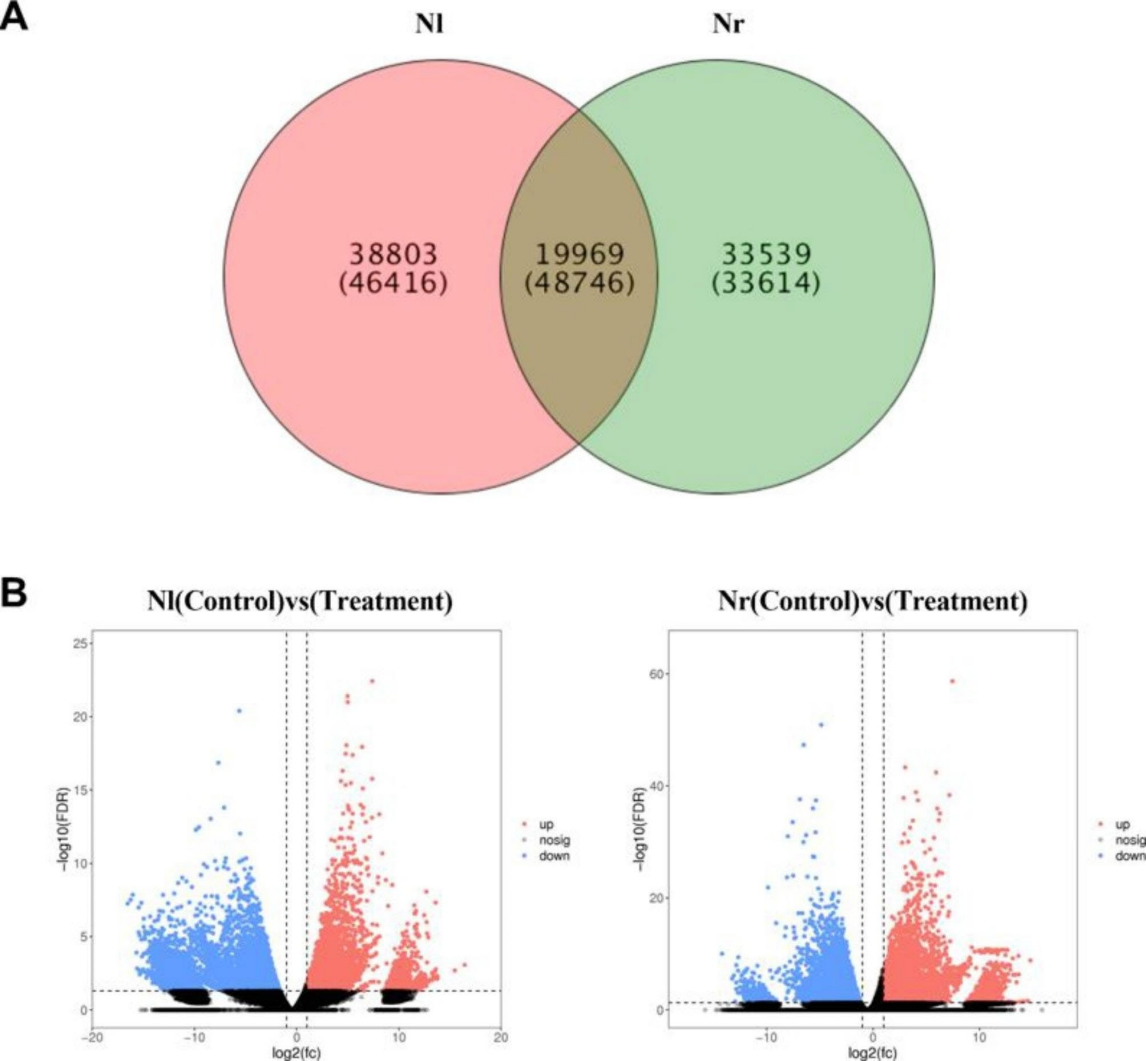
Homologous gene family analysis and DEGs gene expression analysis of Nl and Nr under the cold stress. **A**. Venn diagram of overall statistics of homologous gene families in Nl and Nr. **B**. Difference comparison volcano map. Red (upregulated expression) and blue (downregulated expression) points indicate that there is a difference in gene expression (judgement standard is FDR < 0.05, and the difference multiple is more than twice), and the black point is no difference

Nl and Nr belong to the genus *Nymphaea* of the family *Nymphaeaceae*. Nl is hexaploid, and Nr is octoploid. OrthoMCL was used to cluster 128,776 protein-coding genes, and a total of 92,311 homologous gene families were identified. Nl and Nr have a total of 19,969 homologous gene families, of which 25,392 genes are from Nl and 23,354 genes are from Nr. Nl has 38,803 homologous gene families, including 46,416 genes. Nr has 33,539 homologous gene families, including 33,614 genes (Fig. 3A). DESeq2 was used to identify differentially expressed genes (DEGs) between the cold treatment group and the control group at a specific threshold (FDR < 0.05 and absolute fold change ≥ 2). Compared with the control conditions, Nl and Nr showed significant changes in gene expression under the cold stress, but the number of changed genes was different. In Nl, 14,973 genes were differentially expressed when exposed to cold stress for 24 h. Of the DEGs, 5424 were upregulated, while 9549 genes were downregulated. In Nr, 14,345 genes were differentially expressed when exposed to cold stress for 24 h. Of the DEGs, 7683 were upregulated, while 6662 genes were downregulated. (Fig. 3B).

### Gene ontology (GO) enrichment analysis of DEGs

The results showed that the 6170 DEGs were categorized into 1774 GO terms in Nl. Based on GO enrichment analysis (Q-value ≤ 0.05), we screened for the most significantly enriched GO terms. The primary cellular component terms were ‘ribosome’ (GO:0005840), ‘ribosomal subunit’ (GO:0044391) and ‘nonmembrane-bounded organelle’ (GO:0043228). The primary molecular function terms were ‘structural molecule activity’ (GO:0005198). The results showed that the 5258 DEGs were categorized into 1926 GO terms in Nr. Based on GO enrichment analysis (Q-value ≤ 0.05), we screened for the most significantly enriched GO terms. The primary cellular component terms were ‘intrinsic component of membrane’ (GO:0031224), ‘membrane’ (GO:0016020), ‘plastid’ (GO:0009536), ‘membrane-bounded organelle’ (GO:0043227) and ‘intracellular membrane-bounded organelle’ (GO:0043231). The primary biological processes terms were ‘phosphorus metabolic process’ (GO:0005198) and ‘anion transport’ (GO:0006820).

**Fig. 4:**
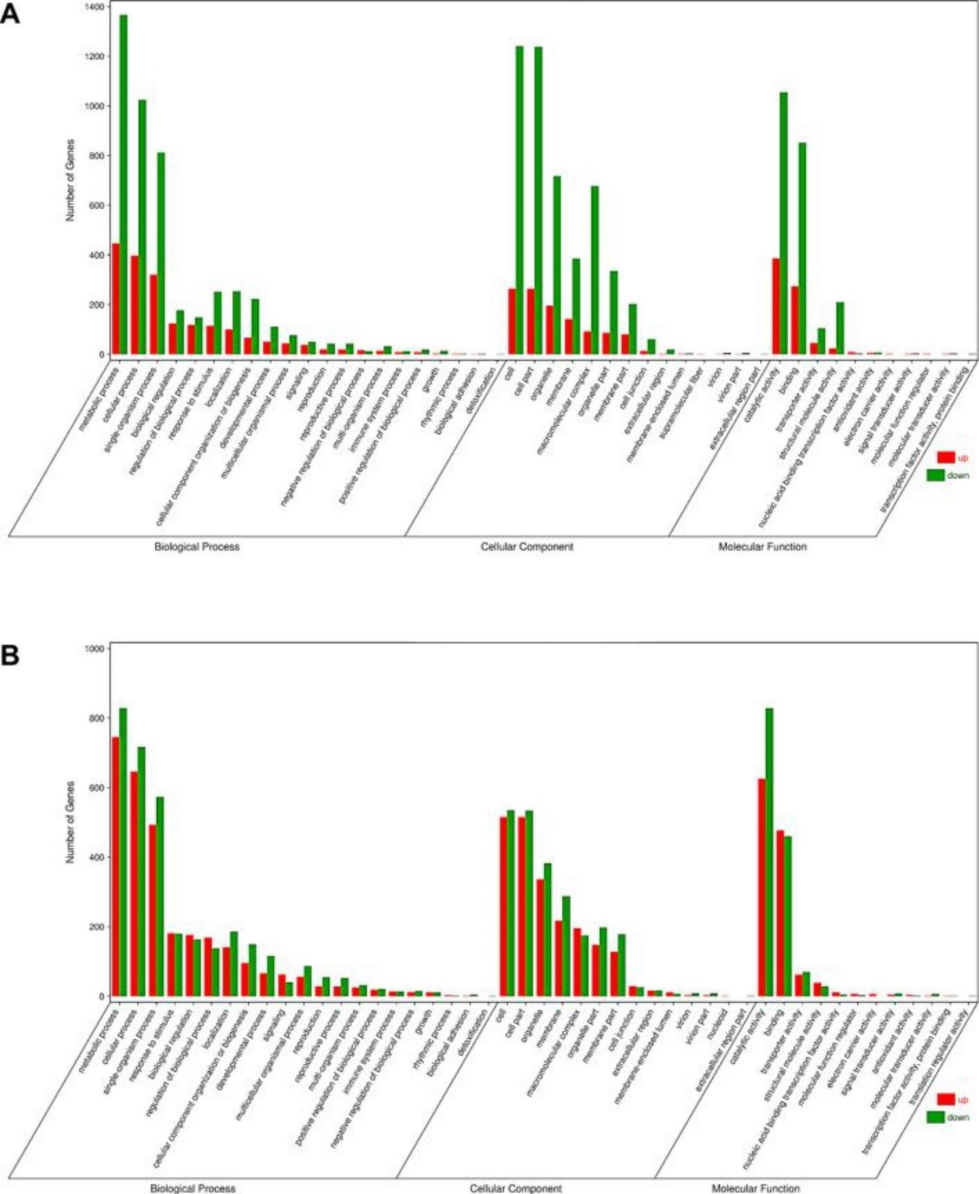
The abscissa is the secondary GO term, the ordinate is the number of genes in the term, red means upregulation, and green means downregulation. **A**. GO enrichment classification histogram of Nl. **B**. GO enrichment classification histogram of Nr

In total, 178 enriched GO terms related to biological processes (BP) were shared by both genotypes. Some GO terms were consistent with the response to cold stress in Nr, including ‘second- messenger-mediated signalling’, ‘phosphorus metabolic process’, ‘detection of stimulus’, ‘response to hormone’ and ‘hydrogen peroxide metabolic process’, which shows that Nr may produce a large quantity of cold signal perception and transmission and cause the metabolism of some cold-resistant substances and oxygenated compounds under the cold stress. Regarding genotype-specific enriched Gene Ontology terms belonging to the biological process (BP) class, some GO terms were enriched in Nl but not in Nr. They are the ‘abscisic acid-activated signalling pathway’, ‘response to abscisic acid stimulus’, ‘phosphorelay signal transduction system’ and ‘response to abscisic acid’, which shows that Nl may transmit signals through abscisic acid hormone to induce the expression of genes related to cold tolerance to achieve the purpose of resisting cold stress. These differences in enriched GO terms between contrasting genotypes indicate that pathways related to these terms could be differentially implicated in response to chilling stress in sensitive and resistant genotypes (Fig. 4).

### KEGG pathway enrichment analysis of DEGs

**Fig. 5:**
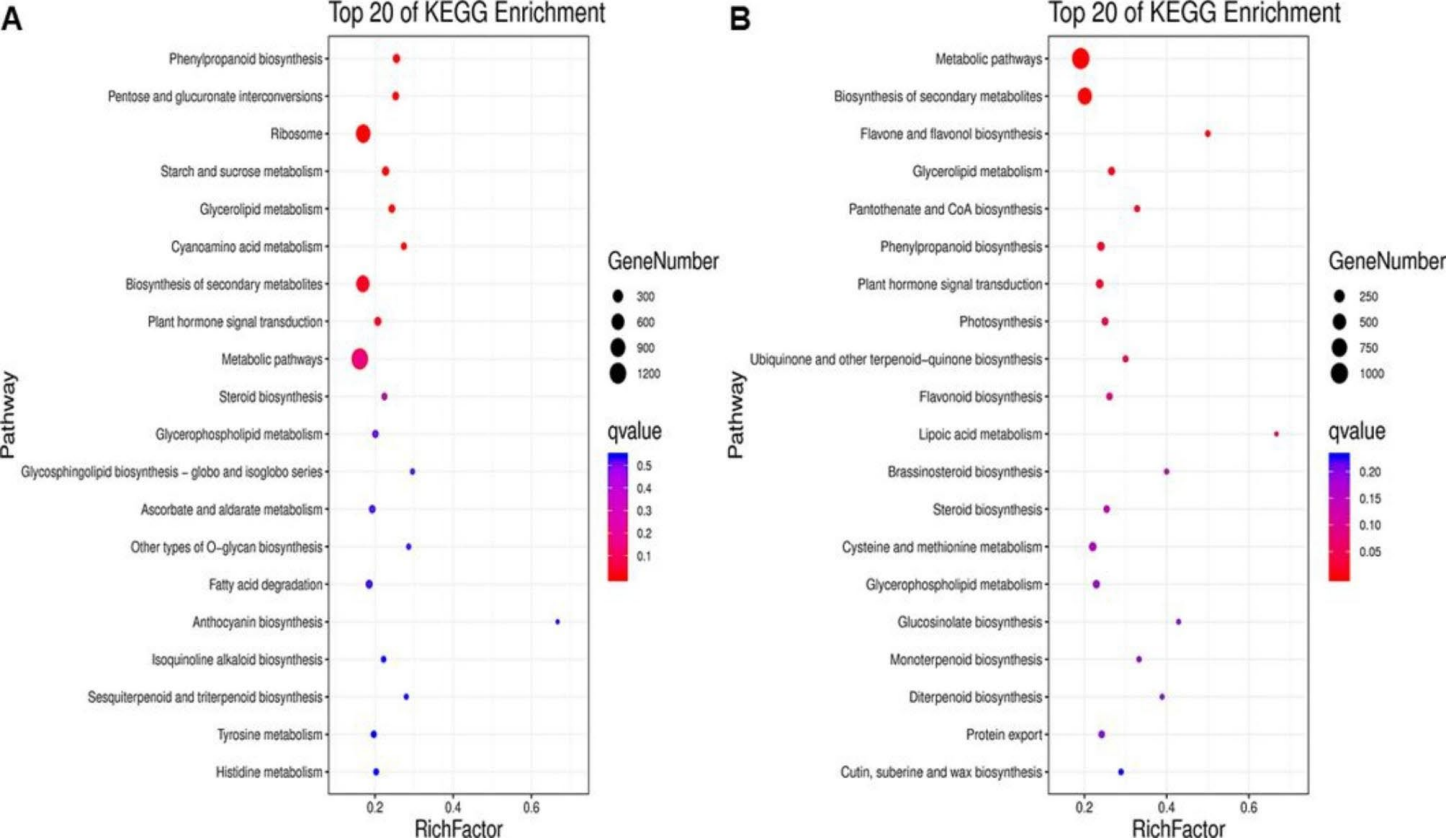
The ordinate is the pathway, and the abscissa is the enrichment factor. The size indicates the number: the redder the colour, the smaller the Q value. **A**. KO enrichment bubble chart of Nl. **B**. KO enrichment bubble chart of Nr

To understand the biological functions of the DEGs, we mapped the DEGs to reference canonical pathways in the KEGG database. The results showed that DEGs from both the Nl and Nr populations were categorized into 14 KEGG pathways (Q-value ≤ 0.05). After 24 h of cold stress, the DEGs from Nl were significantly enriched in ‘Phenylpropanoid biosynthesis’ (ko00940), ‘Starch and sucrose metabolism’ (ko00500), ‘Glycerolipid metabolism’ (ko00561) and ‘Biosynthesis of secondary metabolites’ (ko01110). DEGs from Nr were significantly enriched in ‘Metabolic pathways’, ‘Biosynthesis of secondary metabolites’, ‘Glycerolipid metabolism’, ‘Phenylpropanoid biosynthesis’ and ‘Plant hormone signal transduction’. Comparative analysis of KEGG enrichment in Nl and Nr revealed that genes related to ‘Phenylpropanoid biosynthesis’ and ‘Starch and sucrose metabolism’ have more significant enrichment in Nl. Compared with Nl, genes enriched in ’Metabolic pathways’, ‘Plant hormone signal transduction’ and ‘Biosynthesis of secondary metabolites’ are more significant in Nr. These analysis results show that the two genotypes have some commonality in dealing with cold stress, but the degree of severity may affect their response to cold (Fig. 5).

### Transcription factors involved in cold stress regulation

**Fig. 6:**
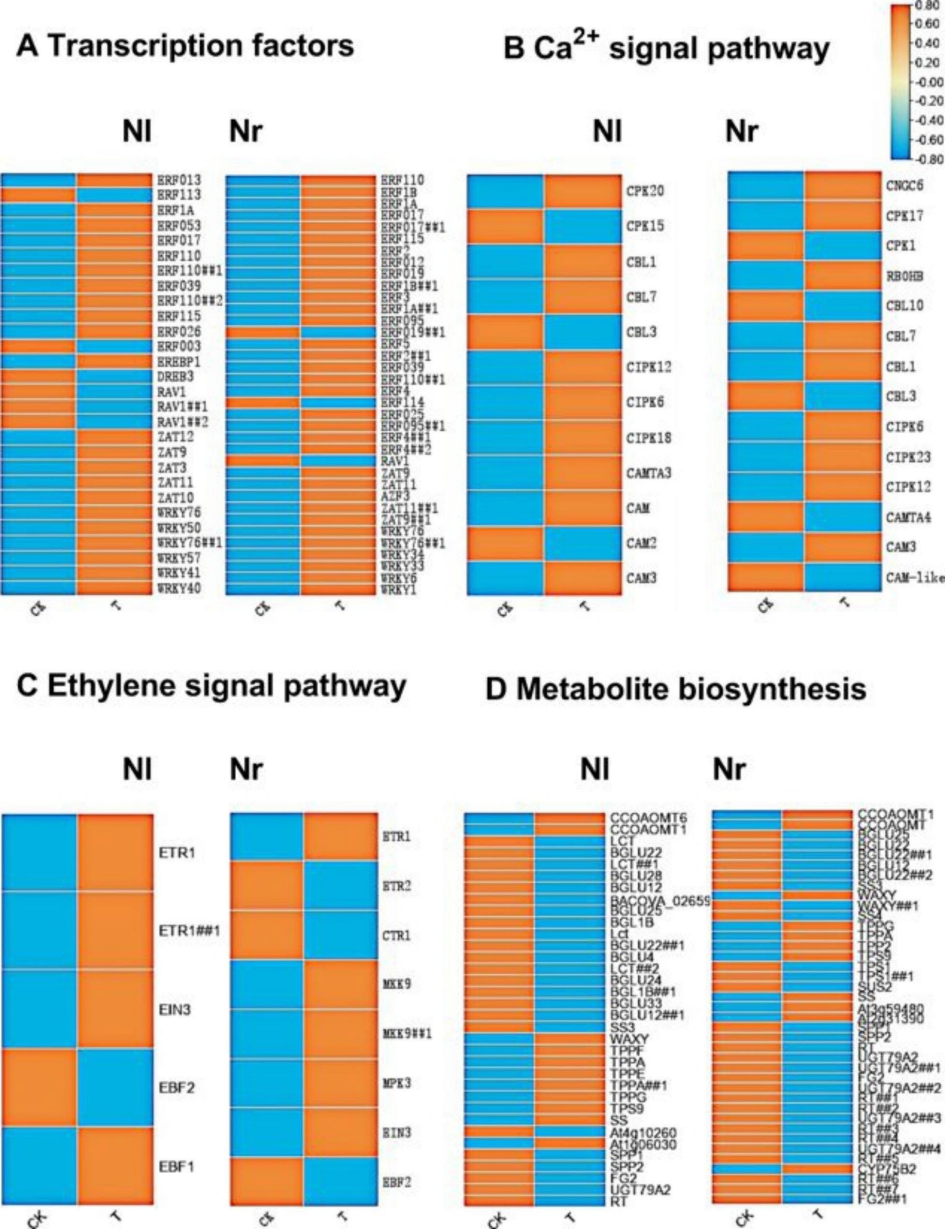
Heatmap analysis of significantly enriched pathways potentially related to cold resistance in Nymphaea. (A) Transcription factor-related genes (B) Calcium ion signalling pathway-related genes (C) Ethylene signalling pathway-related genes (D) Metabolic pathway-related genes

Transcription factors (TFs) play fundamental roles in plant growth and development, as well as in regulating abiotic stress response networks. Under the cold stress, transcription factors regulate upstream cold signals to activate downstream genes associated with cold tolerance. RNA-seq analysis revealed that a large number of TFs are regulated by cold in water lily, and the represented TF families included AP2/ERF, zinc finger, WRKY and MYB. Among these families, 27 and 28 unigenes belonging to the AP2/ERF family were differentially expressed, respectively. Most unigenes were upregulated in both Nl and Nr during cold stress. Among them, ERF110 and ERF2 were significantly upregulated in Nl and Nr. Some unigenes had higher transcriptional levels in Nr than in Nl, such as ERF4, ERF9, and ERF105. In addition, the expression of RAV1 was significantly downregulated in Nl. In Nl and Nr, all genes of the zinc finger family containing the C_2_H_2_ domain were upregulated after cold stress. Among them, ZAT12 and ZAT10 show high transcription level expression in Nl and are stronger than in Nr. ZAT11 had a significantly high level of upregulated expression in both varieties, while ZAT3 and ZAT9 also had significantly upregulated expression, although the expression level was slightly lower. In the WRKY family, WRKY76, WRKY40 and WRKY48 were strongly upregulated in both Nl and Nr during cold stress. In addition, more WRKY genes have been identified in Nr. These data suggested that many TFs could be involved in the positive response mechanism to cold stress (Fig. 6A).

### Calcium signalling and ROS-related genes

Ca^2+^ is a second messenger that plays a key role in the abiotic stress signalling pathway in plants. Changes in calcium channels activate ROS-related genes and cause the production of reactive oxygen species. ROS (reactive oxygen species) are an important signal for plant growth and development and resistance to environmental stress, and their excessive accumulation will cause oxidative stress. The ion transport channel protein CNGC was significantly differentially expressed in Nl and Nr, 7 differentially expressed genes were enriched in Nl, and 2 differentially expressed genes were enriched in Nr. Calcineurin B-like protein (CBL) and CBL-interacting serine/threonine-protein kinase (CIPK) was enriched in Nl and Nr, respectively. The significance of the two genes and the change in transcription level are higher in Nr than in Nl. The CBL and CIPK complex phosphorylates the respiratory burst oxidase homologue protein (RBOH) protein, causing the generation of ROS. In addition, affected by the changes in calcium ion concentration, 11 and 4 calcium-dependent protein kinases with significant differential expression were enriched in Nl and Nr, respectively. In Nl, almost all genes were significantly downregulated, while in Nr, the expression of 3 highly significant genes was upregulated. Respiratory burst oxidase homologue protein, the target gene of these genes, was not enriched in Nl to be significantly differentially expressed, but it was significantly differentially expressed in Nr. These data indicate that the calcium signalling and ROS signalling pathways may play an important role in the response to cold stress in water lilies and that Nr is more sensitive to cold stress than Nl (Fig. 6B).

### MAPK and ethylene signalling pathway-related genes

The MAPK pathway plays an important role in the response to abiotic stress. After cold stress, a whole pathway of ethylene and MAPK coresponse to stress was significantly enriched in Nr. The expression of ethylene receptor ETR1 was upregulated, the negative regulator of ethylene signal CTR1 was downregulated, the expression of mitogen-activated protein kinase kinase 9 (MKK9) was upregulated, the expression of mitogen-activated protein kinase 3 (MPK3) was upregulated, and ethylene insensitive 3 (EIN3) expression was upregulated. The F-BOX protein EBF1/2 that interacts with EIN3 was downregulated, and the target gene ERF1 of EIN3 was upregulated. After cold stress, Nl also responds to ethylene, and some genes were differentially expressed, such as EIN3 and ERF1, but they were significantly lower than Nr. MKK9 and MPK3 also had no significant differential expression in Nl. These data indicate that the ethylene signalling pathway and MAPK signalling pathway may play an important role in the response of water lilies to cold, and Nr may be more sensitive to cold stress (Fig. 6C).

### Metabolite biosynthesis

After the plant undergoes cold stress, the metabolic process will change. Based on the GO and KEGG pathway enrichment analyses, many DEGs are associated with metabolism and biosynthesis. Both Nl and Nr were enriched in a certain number of significantly different genes in ‘Phenylpropanoid biosynthesis’, ‘Glycerolipid metabolism’ and ‘Biosynthesis of secondary metabolite’. In both, the expression of genes related to caffeoyl-CoA O-methyltransferase was significantly upregulated, and all genes related to glucosidase were significantly downregulated. In Nl, we found that 87 DEGs were enriched in the ‘starch and sucrose metabolism’ (ko00500) pathway. The expression of granule-bound starch synthase, trehalose 6-phosphate synthase, sucrose synthase and fructokinase was upregulated after cold stress, and sucrose-6-phosphatase and starch synthase were downregulated. In Nr, we found that 16 DEGs were enriched in ‘Flavone and flavonol biosynthesis’. There were 15 downregulated genes, of which 13 DEGs were related to flavonol-3-O-glucoside L-rhamnosyl-transferase and 2 DEGs were related to flavonol-3-O-glucoside/galactoside glucosyltransferase. Starch, sugars and flavonols are effective cold-resistant substances for plants to cope with cold stress. These data indicate that the metabolic synthesis process may play an important role in the response of water lilies to cold stress (Fig. 6D).

### Fluorescence quantitative verification of key genes in response to cold stress

**Fig. 7:**
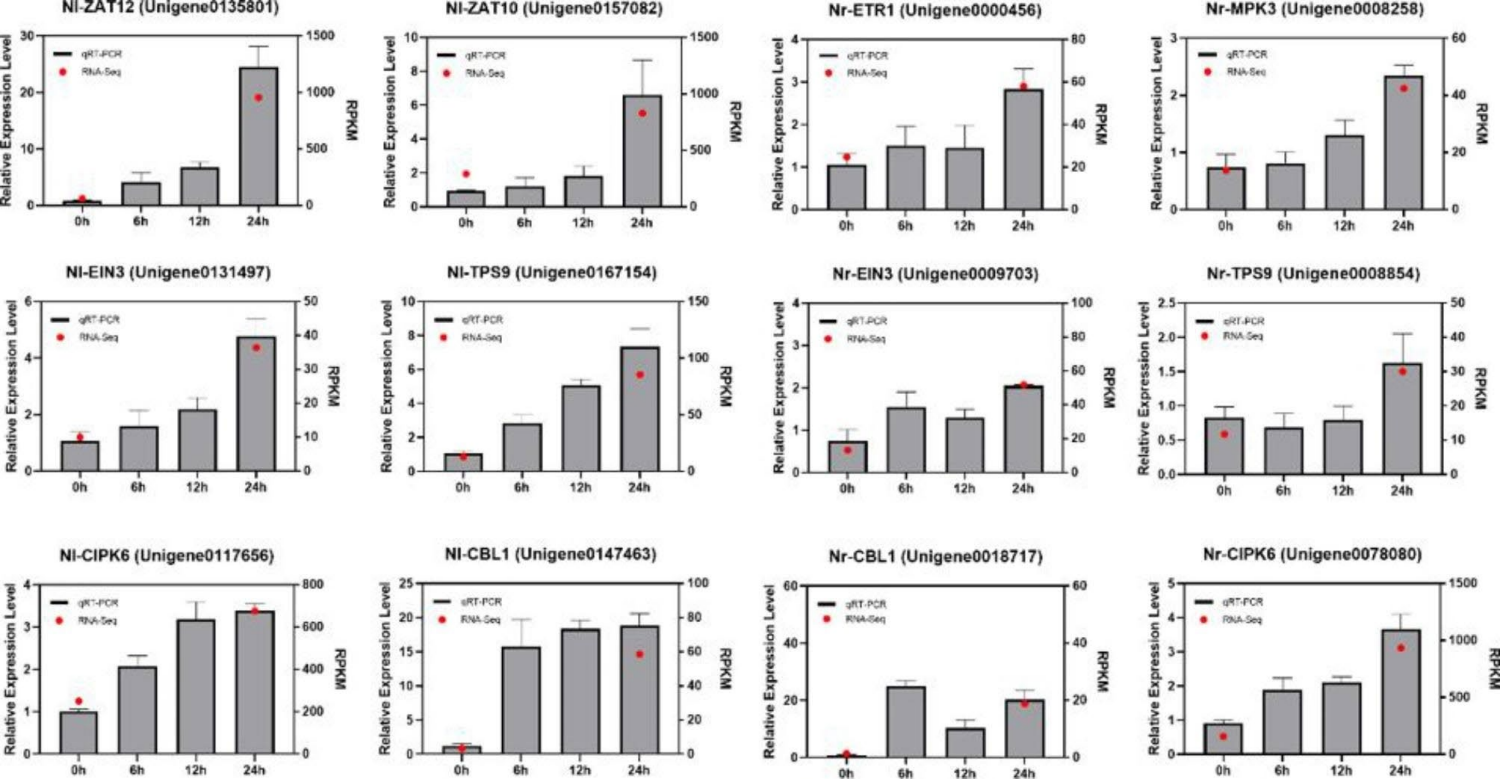
Expression of selected DEGs in response to cold stress by qRT‒PCR. Data are the means ± SDs of three biological replicates. Bars with different letters are significantly different at P < 0.05 (ANOVA followed by Tukey’s post hoc test). Red points were used to describe RNA-Seq.

To confirm the expression levels of DEGs from RNA-Seq data, 12 genes that play a key role in the response to cold stress were selected for transcript abundance analysis and graphing (Fig. 7). In the chart, the real-time fluorescence quantitative data are represented by a bar chart, and the transcriptome expression data are represented by a red dot. The results show that the expression trend of key genes is the same as that of the sequencing data, and the transcriptome data are accurate and reliable. Differentially expressed genes were screened with FDR < 0.05 and |log_2_FC|>1 as criteria. Some of these genes were significantly differentially expressed in Nl but not in Nr, and some genes were significantly differentially expressed in both but at different levels. For example, *ZAT12* had a significant difference in Nl but not in Nr. *ZAT10* was also significantly different in Nl but not Nr. Although *TPS9* is upregulated in both Nl and Nr, *NlTPS9* is more significantly than *NrTPS9.* RPKM values are used to better validate in the plot that the transcriptome data can be in the same trend as the expression data obtained from the quantitative experiments.

### Isolation of the *NlZAT12* gene from Nl

Based on the combined analysis of RNA-Seq data and qPCR validation, the transcription factor ZAT12 showed a clear trend of transcriptional upregulation after being subjected to cold stress, and it was more pronounced in Nl. To verify the role of the *NlZAT12* gene in response to cold stress, we isolated the full-length sequence of the coding region of the *NlZAT12* gene from Nl. The full-length coding region of the gene is 452 bp and encodes 185 amino acids in total. The protein is predicted to have a conserved C_2_H_2_ domain at amino acids 41–120 and belong to the zf-C_2_H_2__6 protein family. The relative molecular mass of the protein is 16282.40, and the isoelectric point is 9.14. Among them, arginine (Arg) and leucine (Leu) accounted for more than all amino acids, accounting for 9.9% and 8.6%, respectively. The molecular formula is C_702_H_1124_N_226_O_210_S_6_; there are 18 negatively charged residues (Asp + Glu) and 23 positively charged residues (Arg + Lys), and the instability coefficient is 66.29. The fat index was 66.29, and the average hydrophilicity index was − 0.545, which was predicted to be a hydrophilic unstable protein.

**Fig. 8:**
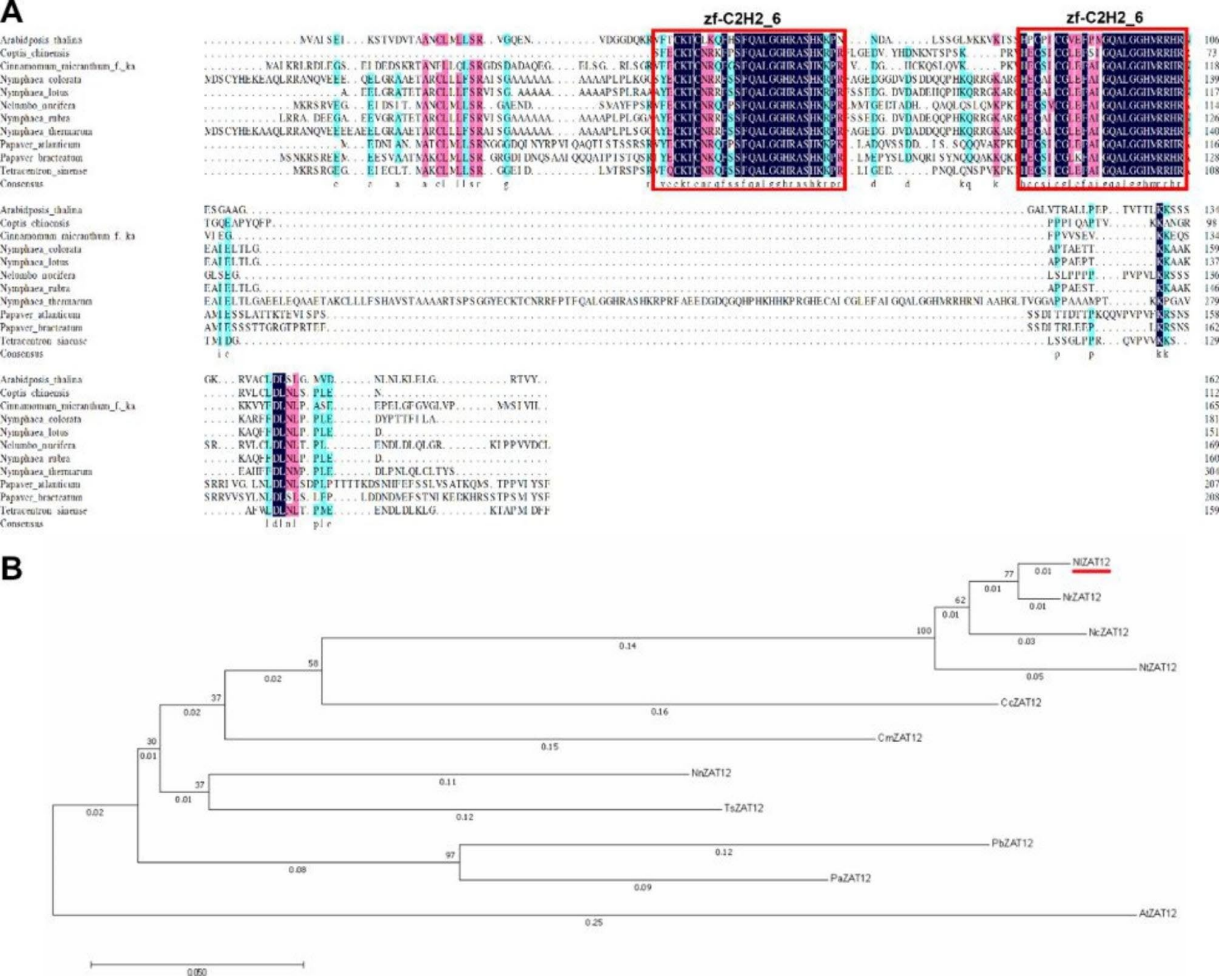
Sequence alignment and phylogenetic relationship between *NlZAT12* and ZAT12 proteins from other species. **(A)** Comparing the alignment of intact *NlZAT12* with other species of ZAT12 proteins, the conserved domains are shown in blue boxes (zf-C2H2_6). **(B)** Phylogenetic tree analysis of *NlZAT12* and ZAT12 proteins from other species

### Phylogenetic tree, sequence analysis and subcellular localization

**Fig. 9:**
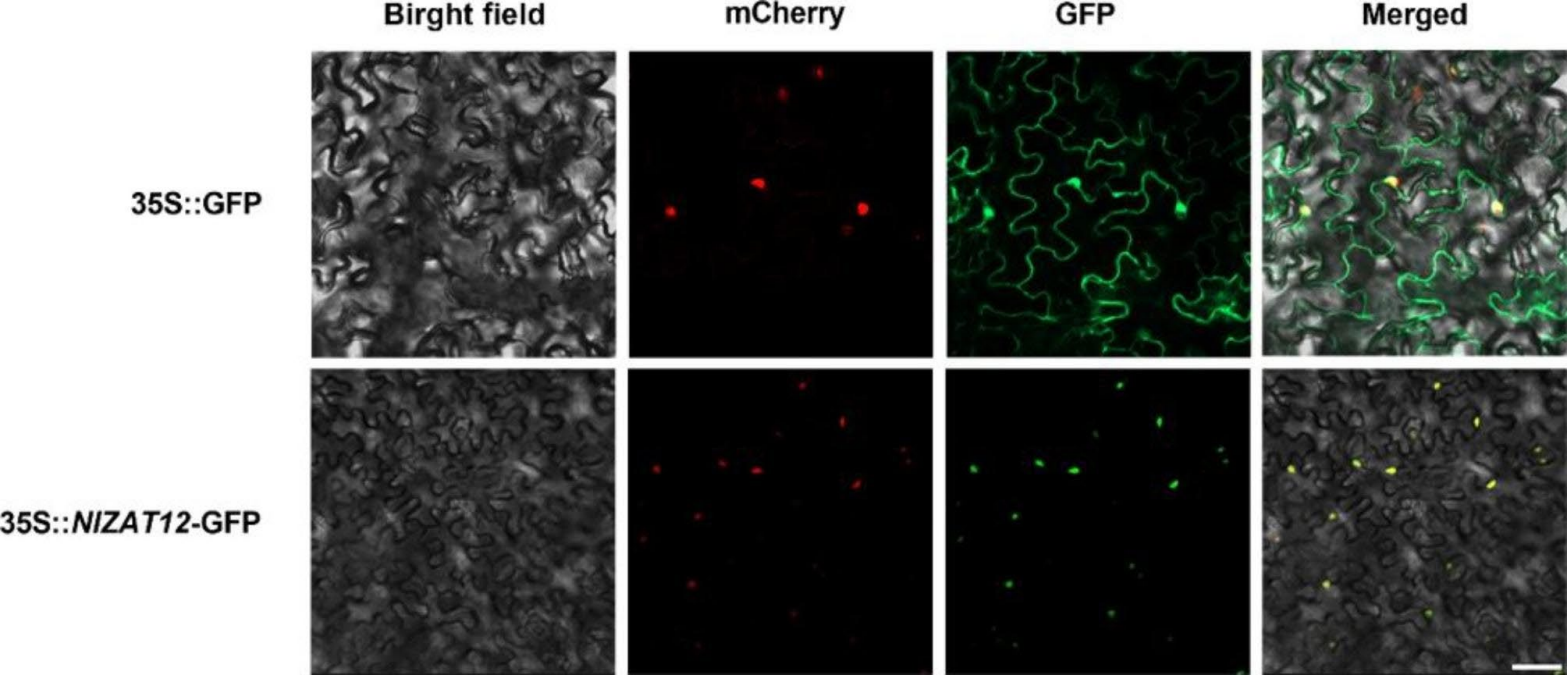
Subcellular localization of *NlZAT12* protein. Subcellular localization of *NlZAT12* protein. The red fluorescence position is the nucleus, the green fluorescence is the GFP, and orange fluorescence is displayed when the two fluorescence positions overlap (bar = 10 μm)

To explore the phylogenetic relationship of the ZAT12 protein in plants, DNAMAN was used to compare the protein sequence of *NlZAT12* with the ZAT12 protein sequence of 10 different species. The NCBI database was searched for *Arabidopsis* (*Arabidposis thalina*), rhizoma coptidis (*Coptis chinensis*), sinkwater camphor (*Cinnamomum micranthum*), bluestar waterlily (*Nymphaea colorata*), lotus (*Nelumbo nucifera*), India red waterlily (*Nymphaea rubra*), rwanda waterlily (*Nymphaea thermarum*), Moroccan poppy (*Papaver atlanticum*), Iranian poppy (*Papaver bracteatum*) and water green tree (*Tetracentron sinense*). The phylogenetic analysis results of these homologous genes are shown in the figure (Fig. 8B). ZAT12 contains a C_2_H_2_ conserved domain, and *NlZAT12* and India red waterlily are in the same branch and are relatively closely related (Fig. 8A). To explore the localization of *NlZAT12* protein in cells, a vector pFAST-R05-*NlZAT12* fused with a green fluorescent protein GFP tag was constructed. As shown, the *NlZAT12*-GFP fusion protein was localized in the nucleus, while the control GFP was distributed in the cell membrane and nucleus, indicating that *NlZAT12* is a nuclear-localized protein (Fig. 9).

### Overexpression of *NlZAT12* in *Arabidopsis thaliana* increased cold tolerance

**Fig. 10:**
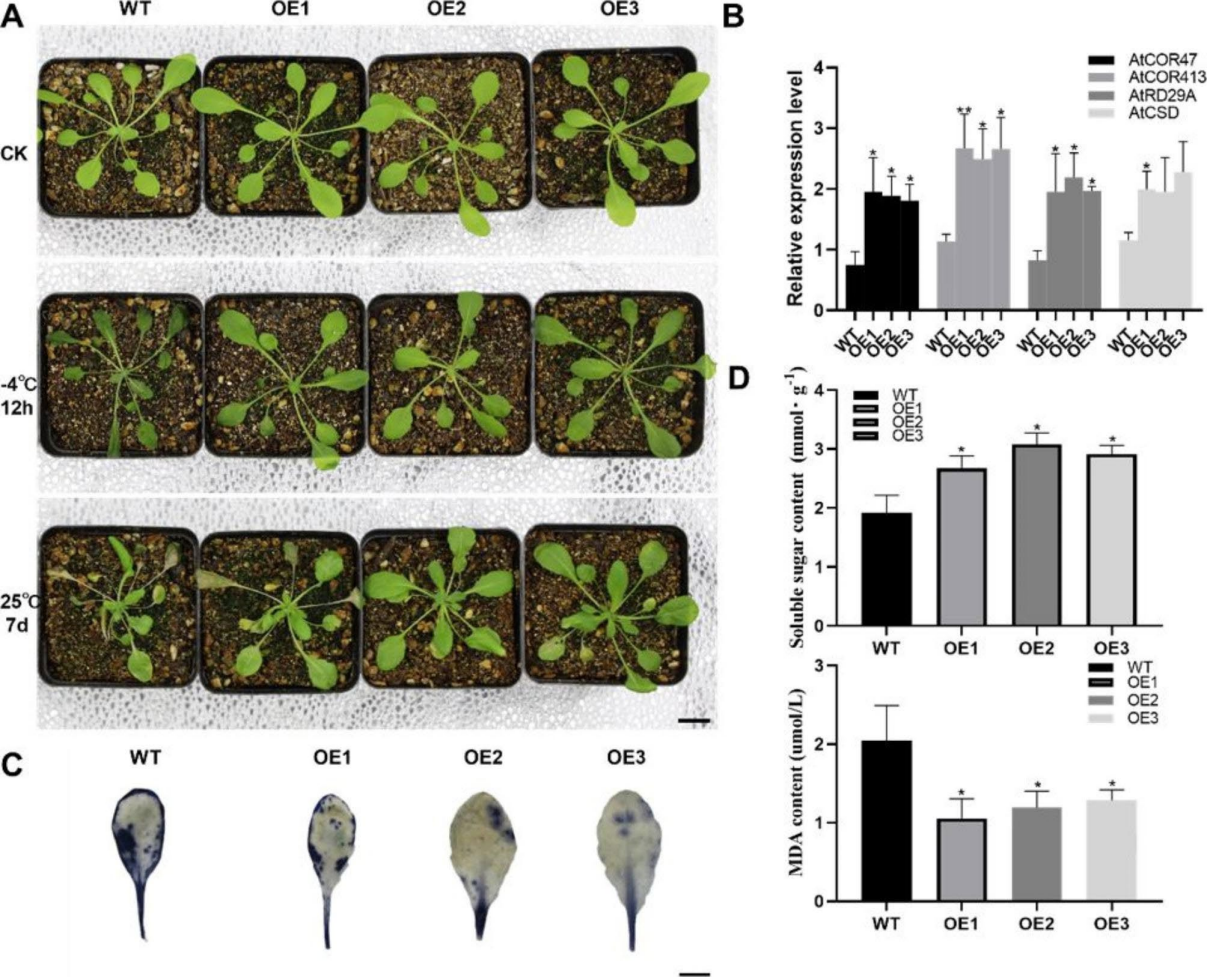
Overexpression of *NlZAT12* in *Arabidopsis* improves the cold tolerance of plants. **(A)** Phenotypes of wild-type and transgenic lines under control (0 h), cold stress (-4 °C, 12 h) and recovery periods (25 °C, 7 d) (bar = 3 cm). **(B)** Relative expression levels of *Arabidopsis thaliana* cold-responsive genes (*AtCOR413*, *AtCOR47*, *AtRD29A*) and antioxidant enzyme-related gene *AtCSD1* in wild-type and transgenic *Arabidopsis thaliana* after cold treatment. Asterisks indicate significance determined by t test: * for p < 0.05 and ** for p < 0.01. **(C)** O_2_^−^ accumulation in the leaves of wild-type and transgenic plants by histochemical staining with NBT after cold treatment (bar = 1 cm). **(D)** Changes in MDA and soluble sugar content of wild-type and transgenic lines after low-temperature treatment. Asterisks indicate significance determined by t test: * for p < 0.05 and ** for p < 0.01. Data are shown as the mean of three independent experiments. These results are based on three biological replicates and three technical replicates

To study the function of *NlZAT12*, the pFAST-R05-*NlZAT12* overexpression vector was constructed, and *Arabidopsis thaliana* was transformed by Agrobacterium infection to obtain 3 healthy transgenic lines (OE1, OE2, OE3) to investigate whether *NlZAT12* is related to cold tolerance. As shown, under control conditions, WT and OE were not significantly different and grew well. After 12 h of cold stress, WT showed more obvious leaf colour deepening and shrinkage than OE, which indicated that OE was less affected by cold stress than WT (Fig. 10A). As shown, under the cold stress, the soluble sugar level of OE was higher than that of WT, and the MDA level of OE was lower than that of WT (Fig. 10D). The accumulation of ROS levels in OE was significantly lower than that in WT after low-temperature treatment (Fig. 10C). ZAT12 regulates the expression of 24 cold-responsive genes (COR), suggesting that ZAT12 may be involved in cold adaptation through a cold-responsive pathway different from the CBF cold-responsive pathway. In addition, the role of the ZAT12 transcription factor may be to help plants cope with oxidative stress caused by cold [42]. Overexpression of the *NlZAT12* gene in *Arabidopsis* increased the expression levels of *AtCOR47*, *AtCOR413*, *AtRD29A* and *AtCSD1* (Fig. 10B). These results indicated that *NlZAT12* enhanced the cold tolerance of plants under the cold stress conditions.

## Discussion

Tropical water lilies have high ornamental value, but overwintering in open fields at high latitudes is difficult. Therefore, the germplasm innovation of cold-resistant tropical water lilies is important in the water lily industry. Plants respond to cold stress by changing their morphological, physical and biochemical properties [22]. Molecular and genetic studies have shown that these changes may include the expression of numerous related genes [23]. It is necessary to explore the molecular mechanism of tropical water lilies in response to cold stress and to further improve varieties through genetic engineering and other technologies. RNA-Seq technology is an effective method to study the mechanism of plant response to cold stress [24]. In this study, the physiological and biochemical characteristics of the two screened water lily cultivars were explored, focusing on the changes in their morphology, soluble substances and antioxidant enzymes under the cold stress. The results showed that there were differences in the response of the two water lily varieties to cold, which could be used to further explore the molecular mechanism of the response to cold stress. Many DEGs were enriched by RNA-Seq technology, indicating that they have a certain response to cold stress. Many DEGs were induced by cold, and the upregulated genes in Nr were more than those in Nl, while in Nl, the downregulated genes accounted for the majority, indicating that Nl and Nr may have different molecular response mechanisms to cold stress. GO enrichment and KEGG pathway analysis showed that calcium signal transduction, ROS-related genes, phytohormone signal transduction and metabolic processes are involved in the response of water lily to cold stress, indicating that many key genes in these processes play a role in regulating water lily to respond to cold stress (Fig. 6).

Under the cold stress, plants can trigger gene expression in multiple signal transduction pathways, thereby activating downstream regulatory pathways related to physiological adaptation [25]. The release of calcium ions in the cytoplasm has a great influence on cold stress [26]. Intracellular Ca^2+^ sensors, such as CIPKs and calcium-dependent protein kinases (CDPKs), respond to increased Nr cytoplasmic Ca^2+^ levels [27, 28]. In the two water lily varieties, a series of calcium ion signal transduction-related genes were activated, such as CAM, CAMTA, CPK, CBL, and CIPK. These calcium-related proteins play key roles as Ca^2+^ sensors and activities downstream in response to cold stress [29]. CBL proteins interact with a group of serine/threonine protein kinases known as CIPKs [30]. The CBL-CIPK pathway is involved in regulating the response of plants to various environmental stresses, including cold, salt and drought stress [31]. In this study, the significance of the two genes and the change in transcription level were higher in Nr than in Nl. The downstream gene RBOH, which is regulated by CBL-CIPK, showed different degrees of significant differences among them. NADPH oxidase located on the plasma membrane, also known as the RBOH protein, provides a very important ROS synthesis pathway [32]. The activation and regulation of RBOH is likely to depend on the Ca^2+^ signal and the phosphorylation of Ca^2+^-activated CPK or CBL-CIPKs [33, 34]. RBOH showed no significant difference in Nl, and the fold change in Nr was higher than that in Nl. This result may be because Nr is more sensitive to calcium ions and Nl has a certain degree of inhibition of RBOH protein expression. Reactive oxygen species (ROS) perform two functions in plants. One is that ROS are considered harmful to plants because their excessive accumulation may cause oxidative stress and, in severe cases, cell damage. On the other hand, a timely and moderate burst of reactive oxygen species is an important signal for plant growth and development and resistance to environmental stress [32]. ROS activate the expression of many transcription factors, such as zinc finger, MAPK, ERF, etc. Among them, ZAT12 is thought to be involved in cold and oxidative stress signalling in *Arabidopsis thaliana*[35]. The ZAT12 gene response to different stresses may be due to the accumulation of reactive oxygen species (such as H_2_O_2_) in the cells that activate the expression of ZAT12 [36, 37]. Under oxidative stress, the gene knockout ZAT12 plants lack the expression of Apx1, the gene knockout plants are highly sensitive to oxidative stress, and the ZAT12 overexpression lines have enhanced tolerance to methyl viologen [38]. In plants overexpressing ZAT12, peroxidase related to the enhanced metabolism of reactive oxygen species is also expressed. APX and peroxidase can reduce the content of active oxygen in plants to a certain extent. Peanut plants overexpressing ZAT12 are more cold-tolerant than wild plants [39]. In this study, ZAT12 protein expression was significantly upregulated in Nl after cold stress, but there was no significant differential expression in Nr. This may be part of the reason why the leaves of Nr show obvious cold and damage caused by the destruction of active oxygen after cold stress (Fig. 6B and C).

The CBF transcription factor, also known as dehydration response element (DRE) binding factor 1 (DREB1) protein, is essential for the cold acclimation of higher plants [7, 40]. When the plant is exposed to nonfreezing cold, the CBF gene is rapidly induced within 15 min, and then the downstream target COR gene is activated, which is called the CBF regulon [40–43]. In *Arabidopsis*, COR genes include COR, cold induction (LTI), dehydration response (RD) and early dehydration induction (ERD) genes. Some of these genes encode key enzymes for osmotic pressure biosynthesis, which increase cold resistance by accumulating cryoprotective proteins and soluble sugars, thereby repairing cold and rigid membranes and stabilizing cell osmotic potential [39]. Various transcriptional activators working in parallel with CBF, including HSFC1, ZAT12, ZF, ZAT10, RAV1, CZF1 and HY5, help regulate the COR gene by bypassing the CBF signal [42, 44, 45]. In this result, ZAT12 and ZAT10 significantly upregulated Nl had some upregulated COR genes, such as ERD7 and ERD15. In Nl, CBF1 is significantly upregulated, although the increase in transcription level is not obvious. This may be because after cold stress, a series of signal transduction processes and physiological and ecological changes occurred in Nl, and eventually, some substances that improve cell osmotic potential and freezing resistance accumulated so that the leaves of Nl did not show obvious freezing damage (Fig. 6A).

Cold will stimulate changes in the amount of ethylene synthesis in plants [46]. The ethylene signal binds to the ethylene receptor ETR1 to inactivate the negatively regulated structural triple response 1 (CTR1, a Raf-like MAPK kinase (MAPKKK)), thereby specifically activating the MKK9-MPK3/6 module, which is responsible for ethylene signal transduction. Phosphorylate stabilizes ethylene insensitivity 3 (EIN3) during the process and promotes EIN3-mediated ethylene signal transduction [47, 48]. EBF2 is activated in the ethylene signal, which destroys the stability of EIN3 in the nucleus and reduces EIN3 accumulation [49]. EIN3, a positive regulator of ethylene, negatively regulates the cold response pathway. As an important transcription factor downstream of the ethylene signalling pathway, EIN3 directly regulates the core factors of the CBF pathway, CBF1, CBF2, and CBF3, inhibits the expression of CBFs, and negatively regulates the low-temperature response of plants [49, 50]. In this study, the ethylene signalling pathway and related genes that specifically activate the MKK9-MPK3/6 module were all significantly expressed in Nr, in which the expression of ETR1 was significantly upregulated, the expression of CTR1 was downregulated, and both MKK9 and MPK3 were significantly upregulated. Ethylene insensitive (EIN3) and ethylene early response factors (ERFs) were also significantly upregulated in Nr. The upregulated expression of EIN3 may be part of the reason why CBFs are inhibited in Nr, so Nr has a weak ability to withstand cold under the cold stress (Fig. 6C).

Metabolite biosynthesis in response to cold stress affects different components of the metabolic biosynthesis process. Several soluble sugars, such as sucrose, glucose, fructose, ribose and trehalose, often accumulate under the cold stress [51]. These sugars can scavenge free radicals and indirectly induce protein synthesis, thereby improving the cold tolerance of plants [52]. The results of this study showed that under the cold stress, the trehalose-6-phosphate synthase gene was significantly upregulated in Nl, but there was no significant difference in Nr. Trehalose is a safe nonreducing disaccharide found in fungi, bacteria and insects [53]. Trehalose plays a role in protecting biological macromolecules (such as membrane proteins), so it is of great significance to the survival of organisms [54]. Flavonoids prevent oxidative damage under abiotic stress by scavenging excess ROS [55]. The World Health Organization has confirmed that the accumulation of flavonoids can protect morning glory from cold damage. Most flavonoids are superior to well-known antioxidants, such as ascorbic acid, but different flavonoid oxidation processes may also be involved in the scavenging of reactive oxygen species [56]. However, the results of the study showed that the expression of most of the flavonol compounds enriched in Nr was significantly downregulated. When a large amount of ROS is generated in plant cells, Nr may not be cleared quickly and cause ROS accumulation, ultimately damaging plant tissues (Fig. 6D).

**Fig. 11:**
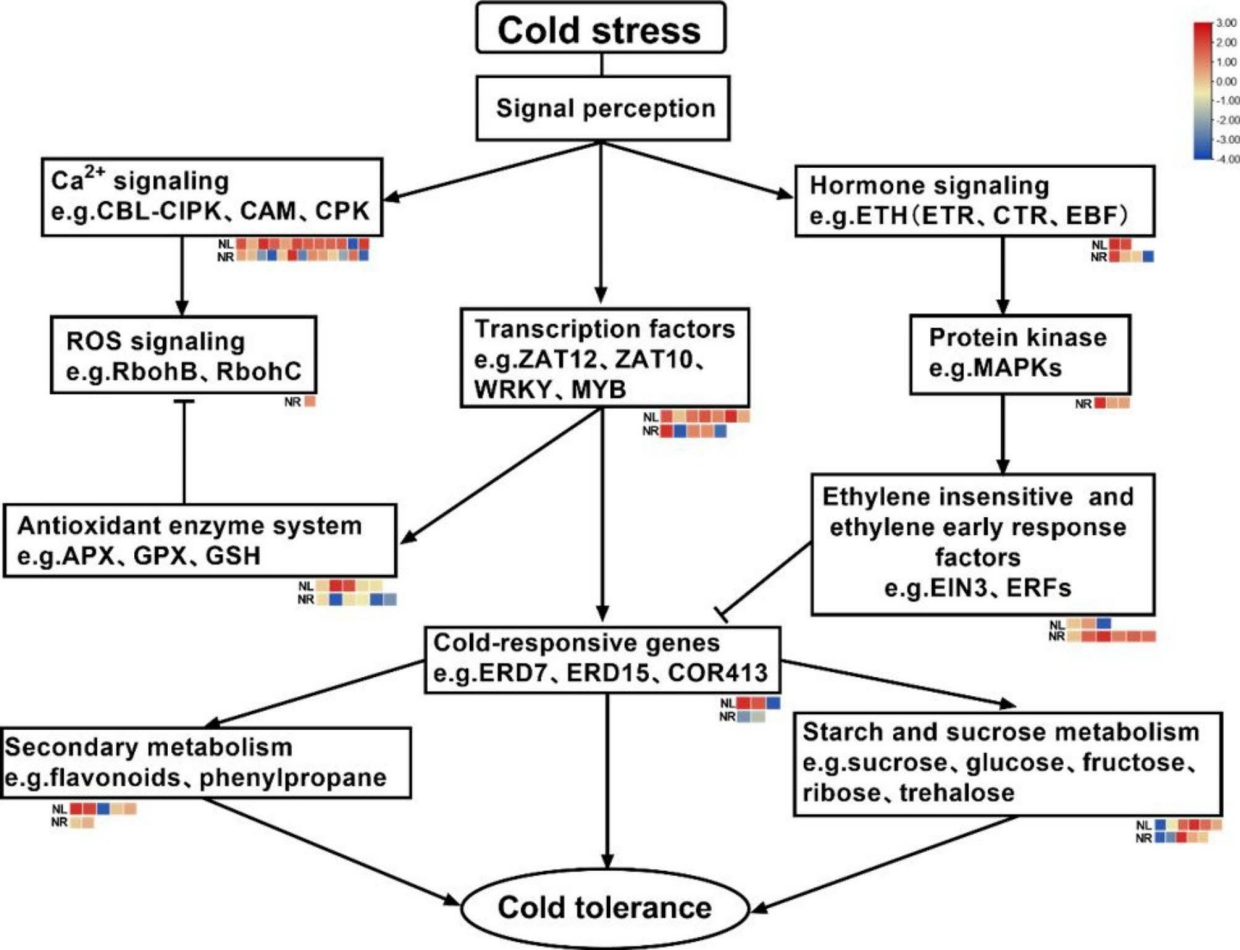
Hypothetical model of the molecular mechanism of the water lily response to cold stress. Calcium signals, transcription factors and hormone signal transduction are important pathways by which water lily responds to cold stress. Heatmap provides global transcriptome regulation of genes related to cold stress in response. Lines with arrows indicate positive regulation, lines with blunt tips indicate negative regulation

Based on the above results, we propose two hypothetical models of water lilies in response to cold stress at the transcriptional level. When cold stress occurs, the water lily leaves first sense the low-temperature signal, which will cause changes in the concentration of intracellular calcium ions, the content of plant hormones (such as ethylene and abscisic acid), and the expression of some transcription factors in the nucleus. The complex formed by the interaction of CBL and CIPK phosphorylates the RBOH protein, causing an increase in the intracellular ROS content, and the accumulation of ROS to a certain extent causes irreversible damage to the cell structure. In Nl, the expression level of the zinc finger family protein ZAT12 increases significantly after cold stress, which can cause the expression of intracellular antioxidant enzyme system genes to eliminate the ROS burst caused by cold stress and maintain the intracellular reactive oxygen species balance. The ZAT12 protein also activates the expression of a large number of cold-responsive genes, which play a key role in improving plant cold tolerance. For example, the early dehydrated protein ERD7 can avoid damage to the cell membrane structure in the early stage of cold stress, and some other cold-responsive genes are also affected to varying degrees. Regulates the expression of starch, carbohydrate and secondary metabolite synthesis genes. In Nr, more genes of the ethylene signal transduction pathway were significantly expressed, and changes in ethylene content caused upregulation of ethylene receptor ETR and downregulation of CTR, a negative regulator of ethylene signalling. At the same time, genes related to protein kinase MAPKs were also significantly expressed in Nr, activating the downstream ethylene signalling transcription factor EIN3 and many ethylene responses to factors ERFs. Among them, EIN3 and some ERF factors can inhibit the expression of cold-responsive genes, which may be one of the reasons why Nr is more sensitive to cold stress (Fig. 11). The names and log_2_FC of differentially expressed genes have been added to additional file (Additional file 6).

Analysis of RNA-Seq data showed that the transcript level of *NlZAT12* in Nl was significantly upregulated after cold stress. Studies have shown that ZAT12 plays a key role in the response to cold stress and oxidative stress [35, 57]. A study by Rizhsky et al. found that ZAT12-overexpressing plants showed limited improvement in cold tolerance [38], while another study showed that *Arabidopsis* lines overexpressing ZAT12 exhibited higher cold tolerance [43]. This difference may be caused by the differential expression of different ZAT12 genes in different plants. We were interested in whether overexpressing *NlZAT12* could result in higher cold tolerance under the cold stress. Protein sequence alignment and phylogenetic tree analysis showed that *NlZAT12* is a member of the zinc finger protein family, and ZAT12 proteins of different species have a conserved zf-C_2_H_2_6_ domain in the middle region (Fig. 8). It can be demonstrated that ZAT12 factors are conserved during evolution and are widely present in plants. The *NlZAT12* protein is localized in the nucleus and may function as a transcription factor in plants (Fig. 9). Under the cold stress, the soluble sugar content of the transgenic lines was significantly higher than that of the wild type, and the MDA content of the transgenic lines was significantly lower than that of the wild type (Fig. 10D). The phenotypes of the wild-type and transgenic lines were also significantly different, and the growth state of the transgenic lines after cold stress was significantly better than that of the wild-type. The production of reactive oxygen species can be caused by various abiotic stresses and plays a key role in responding to stresses [36, 58]. Overexpression of *NlZAT12* reduced the accumulation of ROS in *Arabidopsis* leaves under the cold stress, suggesting that *NlZAT12* may be involved in the regulation of oxidative stress (Fig. 10C). The cold stress response is a complex process involving many cold response genes, such as RD29A and COR47, which play important roles in the regulation of the cold stress response [59]. In this study, the expression levels of the *AtCOR413*, *AtCOR47*, *AtRD29A*, and *AtCSD1* genes were significantly upregulated in the transgenic lines overexpressing *NlZAT12* compared to the wild type (Fig. 10B). This proves that *NlZAT12* may participate in the regulation of cold stress by increasing the expression of cold-responsive proteins.

## Conclusion

In this study, high-throughput sequencing technology was used to sequence the transcriptomes of two water lilies under the cold stress. According to KEGG enrichment analysis, it was found that transcription factors, secondary metabolism, plant hormone signal transduction and reactive oxygen species signal transduction systems. A large number of genes in the related pathways are actively involved in the response to cold stress. Nr enriched more significantly differentially expressed genes in the ethylene signalling pathway and reactive oxygen species signalling pathway, and Nl enriched more differentially expressed genes in response to cold-related transcription factors and metabolites, indicating that the difference in transcription level between the two tropical water lilies may be one of the reasons why they are affected by cold stress.

The key candidate gene *NlZAT12*, which regulates the response of water lily to cold stress, was screened out, and *NlZAT12* was located in the nucleus. Through transgenic experiments, it was found that heterologous overexpression of the *NlZAT12* gene in *Arabidopsis thaliana* can enhance the tolerance of plants to cold stress. This indicates that the *NlZAT12* gene plays an important role in regulating the process of the plant response to cold stress.

## Materials and methods

### Plant material preparation method

In this study, two tropical water lily varieties were selected, namely, cold-tolerant water lily Nl and cold-sensitive water lily Nr. They were kept in the Water Lily Germplasm Resource Garden of Nanjing Agricultural University. When the water lily grows to have 4–5 mature floating leaves, it is exposed to 0 °C temperature after 7 days of low-temperature exercise at 10 °C. No treatment at 25 °C was used as a control. After 24 h of treatment, the leaves of the plants in the treatment group and the control group at the same position were quickly frozen in liquid nitrogen and stored in an ultracold refrigerator at -80 °C for subsequent experiments, and each group had 3 replicates.

Wild-type *Arabidopsis thaliana* seeds Col-0 were sown into MS-resistant medium, and the seeds were preserved at the Aquatic Flower Research Center of Nanjing Agricultural University. To make the germination time of each line as consistent as possible, the Petri dishes were placed in 4 °C cold treatment for more than 3 days after sowing. Seedlings with consistent germination times were transferred to new medium one week after germination. Two weeks after germination, the plants were transplanted into soil for culture (vermiculite: substrate: perlite = 7:3:1).

### Physiological and biochemical index determination method

The method of Lutts et al. was used to determine the relative electrolyte conductivity (REC) of plants in the control and treatment groups [60]. The chlorophyll content was measured using ethanol extraction colorimetry [61]. The thiobarbituric acid (TBA) method was used to determine the malondialdehyde (MDA) content [62]. Superoxide dismutase (SOD) was determined by the nitrogen blue tetrazolium (NBT) method [63]. The method of Xu et al. was used to measure catalase (CAT) activity [64].

### RNA extraction, library construction and sequencing

Total RNA was extracted using a TRIzol reagent kit (Invitrogen, Carlsbad, CA, USA) according to the manufacturer’s protocol. RNA quality was assessed on an Agilent 2100 Bioanalyzer (Agilent Technologies, Palo Alto, CA, USA) and checked using RNase free agarose gel electrophoresis. After total RNA was extracted, eukaryotic mRNA was enriched by Oligo(dT) beads. Then, the enriched mRNA was fragmented into short fragments using fragmentation buffer and reverse transcribed into cDNA by using the NEB Next Ultra RNA Library Prep Kit for Illumina (NEB #7530, New England Biolabs, Ipswich, MA, USA). The purified double-stranded cDNA fragments were end repaired, and A bases were added and ligated to Illumina sequencing adapters. The ligation reaction was purified with AMPure XP Beads (1.0X). Ligated fragments were subjected to size selection by agarose gel electrophoresis and polymerase chain reaction (PCR) amplification. The resulting cDNA library was sequenced using an Illumina Hiseq™ 4000 by Gene Denovo Biotechnology Co. (Guangzhou, China).

### De novo assembly, sequence annotation and protein family analysis

To obtain high quality, FASTp (version 0.18.0) was used to further filter the original data, removing reads containing adapters, reads containing more than 10% of unknown nucleotides (N), and low-quality reads containing more than 50% of low quality (Q-value ≤ 20) bases [65]. The clean reads were employed for de novo assembly using Trinity software, and then the transcripts were clustered and further assembled according to paired-end information to obtain the unigenes [66]. Basic annotation of unigenes includes protein functional annotation, pathway annotation, COG/KOG functional annotation and Gene Ontology (GO) annotation. To annotate the unigenes, we used the BLASTx program with an E-value threshold of 1e-5 to the NCBI nonredundant protein (Nr) database, the Swiss-Prot protein database, the Kyoto Encyclopedia of Genes and Genomes (KEGG) database, and the COG/KOG database [67]. The protein sequences were aligned in pairs, and in the blastp alignment results, similar proteins were identified with an E value less than 1e-7. Subsequently, OrthoMCL was used to group genes with similar sequences into the same family, and finally obtain homologous and unique genes and gene families between species.

### Analysis and functional enrichment of DEGs

The gene abundances were calculated and normalized to RPKM (reads per kb per million reads). The RPKM method is able to eliminate the influence of different gene lengths and sequencing data amounts on the calculation of gene expression. Therefore, the calculated gene expression can be directly used for comparing the difference in gene expression among samples. RNA differential expression analysis was performed by DESeq2 software between two different groups [68]. Genes with a false discovery rate (FDR) below 0.05 and absolute fold change ≥ 2 were considered differentially expressed genes. GO enrichment analysis provides all GO terms that are significantly enriched in DEGs compared to the genome background and filters the DEGs that correspond to biological functions. First, all DEGs were mapped to GO terms in the Gene Ontology database, gene numbers were calculated for every term, and significantly enriched GO terms in DEGs compared to the genome background were defined by a hypergeometric test. The calculated p value was subjected to FDR correction, taking FDR ≤ 0.05 as a threshold. GO terms meeting this condition were defined as significantly enriched GO terms in DEGs. KEGG is the major public pathway-related database [69]. Pathway enrichment analysis identified significantly enriched metabolic pathways or signal transduction pathways in DEGs compared with the whole genome background. The calculation formula is the same as that used in the GO analysis. The calculated p value was subjected to FDR correction, taking FDR ≤ 0.05 as a threshold. Pathways meeting this condition were defined as significantly enriched pathways in DEGs.

### Quantitative real-time PCR validation

To verify the reliability and accuracy of the RNA-seq data, twelve genes were selected for validation by qRT‒PCR. Primers are added in additional file (Additional file 7). Refer to the RNA extraction, library construction and sequencing subsections for total RNA extraction and reverse transcription steps. qRT‒PCR was performed with the Step One Plus™ Real-Time PCR System (ABI) using SYBR premix Ex Taq (Takara, Japan). Each experiment included three biological replicates. The reaction conditions were 94 °C for 5 min, followed by 40 cycles of 95 °C for 15 s and 60 °C for 1 min. The relative level of gene expression was calculated using the 2-ΔΔct formula. The specific primers were designed with Primer software (v 6.24, Primer, Quebec City, Canada). qRT‒PCR analysis included three independent technical repeats with three biological replicates.

### Isolation and subcellular localization of *NlZAT12*

Refer to the RNA extraction, library construction and sequencing subsections for total RNA extraction and reverse transcription steps. Using the first cDNA of Nl as a template, a pair of primers (*NlZAT12*-F and *NlZAT12-*R) were designed according to the CDS region of *NlZAT12*, and the full-length coding region sequence of *NlZAT12* was obtained by PCR. The reaction conditions were 98 °C for 2 min, followed by 35 cycles of 98 °C for 10 s, 60 °C for 20 s, and 72 °C for 30 s, followed by 72 °C for 5 min to obtain the DNA product. The obtained DNA fragments were purified by gel electrophoresis, ligated into the pFAST-R05-GFP vector using homologous recombination, and sequenced.

The open reading frame (ORF) of *NlZAT12* was inserted into pFAST-R05-GFP to construct a fusion vector, which was transformed into Agrobacterium GV3101. By Agrobacterium transformation, the bacterial solution containing pFAST-R05-*NlZAT12* was injected into nuclear-localized transgenic tobacco leaves approximately 3 weeks old. After injection, the tobacco was cultured in the dark for 24 h and then transferred to a long-day (light/dark = 16/8 h) culture room for 2 days. mCHERRY red was used as a marker for nuclei, and the expression of *NlZAT12*-GFP was observed by confocal microscopy.

### Heterologous overexpression of *NlZAT12* in *Arabidopsis thaliana*

After flowering, the infection solution containing the pFAST-R05-*NlZAT12* overexpression fusion vector (containing the RFP fragment) was dipped into the *Arabidopsis* bud. After the fruit pods were mature, the seeds of T0 generation *Arabidopsis* were harvested, and the seeds with the best expression effect were screened under a stereoscopic fluorescence microscope. The above process was repeated twice to obtain T2 generation seeds. The T2 generation seeds were cultivated in the same way.

When they grew to 8–10 leaves, the wild-type and transgenic lines were placed together in a 4 °C incubator for cold acclimation, and after 3 days, they were placed in a -4 °C incubator for low-temperature treatment. The *Arabidopsis thaliana* leaves were then quickly frozen in liquid nitrogen and placed in a -80 °C ultracold freezer for the subsequent determination of gene expression and physiological and biochemical indicators.

## Electronic supplementary material

Below is the link to the electronic supplementary material.

**Additional file 1**.

**Additional file 2**.

**Additional file 3**.

**Additional file 4**.

**Additional file 5**.

**Additional file 6**.

**Additional file 7**.

**Additional file 8**.

**Additional file 9**.

**Additional file 10**.

**Additional file 11**.

**Additional file 12**.

## Data Availability

The datasets generated or analysed during this study are included in this published article and its additional files. Raw Illumina sequence data were deposited in the National Center for Biotechnology Information (NCBI) and accessed in the sequence read archive (SRA) database (https://www.ncbi.nlm.nih.gov/sra). The accession number is PRJNA598031 (https://www.ncbi.nlm.nih.gov/bioproject/PRJNA833097), which includes 12 accession items (SAMN27959287-SAMN27959298).

## Abbreviations

DEGs: Differentially expressed genes
qRT‒PCR: Quantitative real-time PCR
NR: NCBI nonredundant protein database
COG: Clusters of Orthologous Groups
RPKM: Reads Per Kilobase per Million mapped reads
KEGG: Kyoto Encyclopedia of Genes and Genomes
GO: Gene Ontology
Blast: Basic Local Alignment Search Tool
MAPK: Mitogen-activated protein kinase
ABA: Abscisic acid
CDS: Coding sequence
ROS: Reactive oxygen species
